# Erratum to “Associations of Cardiorespiratory Fitness and Fatness with Metabolic Syndrome in Rural Women with Prehypertension”

**DOI:** 10.1155/2014/412430

**Published:** 2014-04-28

**Authors:** Patricia A. Hageman, Carol H. Pullen, Melody Hertzog, Linda S. Boeckner, Susan Noble Walker

**Affiliations:** ^1^Division of Physical Therapy Education, School of Allied Health Professions, College of Medicine, University of Nebraska Medical Center, Omaha, NE 68198-4420, USA; ^2^College of Nursing, University of Nebraska Medical Center, Omaha, NE 68198-5330, USA; ^3^College of Nursing, University of Nebraska Medical Center, Lincoln, NE 68588-0220, USA; ^4^Nutrition and Health Sciences, University of Nebraska-Lincoln, Lincoln, NE 68583-0806, USA

In the original paper, the authors discovered a computer coding error that resulted in 33 of the women's ages being incorrectly recorded.

All analyses were repeated for this paper using the corrected age dataset, as all our logistic regression analyses in the published paper were adjusted for age. The repeated analyses, using the corrected dataset, lead to minor changes that needed to be reported to the results in the published paper. These corrections did not change the conclusion of the published paper. The authors apologize for any inconvenience.

*In the Abstract.* line 8, should be changed from 7% to 8%; line 9, should be changed from 75% to 81%; line 9, should be changed from 59% to 69%.

Tables 2-3 were updated as provided.

*In Section 2.1*. page 3, column 1, paragraph 2, line 1, 57.8 ± 7.6 should be changed to 56.4 ± 6.3.

*In Section 2.2*. page 3, column 1, paragraph 1, line 5, 70 should be changed to 170.

*In Section 3*. page 4, column 2, paragraph 2, line 5, 1.21 should be changed to 1.20; in line 5, 1.14–1.29 should be changed to 1.13–1.27; in line 8, 7% should be changed to 8%; in line 9, 0.93 should be changed to 0.92; in line 9, 0.90–0.97 should be changed to 0.88–0.96; in line 10, = 0.001 should be changed to < 0.001.

*In Section 3*. page 4, column 2, paragraph 3, line 5, 75% should be changed to 81%; in line 6, 0.25 should be changed to 0.19; in line 6, 0.12–0.52 should be changed to 0.09–0.41; in line 10, 59% should be changed to 69%; in line 10, 0.41 should be changed to 0.31; in line 10, 0.19–0.87 should be changed to 0.14–0.69; in line 10, 0.02 should be changed to 0.004.

## Figures and Tables

**Table 2:** Logistic regressions predicting the metabolic syndrome from percent body fat and estimated cardiorespiratory fitness.

	Model 1				Model 2				Model 3			
	*b *	OR	95% CI	*P *	*b *	OR	95% CI	*P *	*b *	OR	95% CI	*P *
% Body fat	0.18	1.20	1.13–1.27	<0.001	0.20	1.22	1.13–1.31	<0.001	0.07	1.07	0.88–1.30	NS
Fitness					0.02	1.02	0.96–1.08	NS	−0.24	0.78	0.53–1.15	NS
% Body fat × fitness									0.01	1.01	1.00-1.01	NS

Fitness	−0.09	0.92	0.88–0.96	<0.001								

All models were adjusted for age, education, and household income.

Nagelkerke *R* squares were 0.24, 0.24, and 0.25 for % body fat models 1, 2 and 3, respectively.

Nagelkerke *R* square was 0.11 for fitness model 1.

**Table 3:** Logistic regressions predicting metabolic syndrome by unfit/fat and fit/fat categories using two body composition methods.

	Cases	*b *	OR	95% CI	*P *
	Model 1—fit/fat categories defining obesity by percent body fat				
Unfit/fat	78/188 (41.5%)		1		
Fit/fat	12/85 (14.1%)	−1.64	0.19	0.09–0.41	<0.001

	Model 2—fit/fat categories defining obesity by revised BMI cut-score				
Unfit/fat	78/179 (43.6%)		1		
Fit/fat	12/57 (21.1%)	−1.17	0.31	0.14–0.69	0.004

“Fat” was defined as body fat cut-score ≥30% and as ≥25 kg/m^2^ for revised BMI obesity cut-score.

“Fit” was >25 mL/kg/min. Both models were adjusted for age, education, and household income.

Both models excluded women classified as “not-fat” as there were no cases of metabolic syndrome in women classified as “non-fat.”

Nagelkerke *R* square was 0.13 and 0.07 for models 1 and 2, respectively.

